# Formulation and *In Vitro* Penetration Study of Recombinant Human Epidermal Growth Factor-Loaded Transfersomal Emulgel

**DOI:** 10.34172/apb.2020.070

**Published:** 2020-08-09

**Authors:** Silvia Surini, Astried Leonyza, Chang Woo Suh

**Affiliations:** ^1^Laboratory of Pharmaceutics and Pharmaceutical Technology Development, Faculty of Pharmacy, Universitas Indonesia, Depok, West Java, 16424, Indonesia.; ^2^PT Daewoong Pharmaceutical Company Indonesia, Jakarta 10230, Indonesia.

**Keywords:** Emulgel, Epidermal growth factor, Penetration study, Percutaneous administration, Transfersomes

## Abstract

***Purpose:*** Recombinant human epidermal growth factor (rhEGF) is a 6045-Da peptide that promotes the cell growth process, and it is also used for cosmetic purposes as an anti-aging compound. However, its penetration into skin is limited by its large molecular size. This study aimed to prepare rhEGF-loaded transfersomal emulgel with enhanced skin penetration compared with that of non-transfersomal rhEGF emulgel.

***Methods:*** Three transfersome formulations were prepared with different ratios between the lipid vesicle (phospholipid and surfactant) and rhEGF (200:1, 133:1, and 100:1) using a thin-film hydration-extrusion method. The physicochemical properties of these transfersomes and the percutaneous delivery of the transfersomal emulgel were evaluated. Long-term and accelerated stability studies were also conducted.

***Results:*** The 200:1 ratio of lipid to drug was optimal for rhEGF-loaded transfersomes, which had a particle size of 128.1 ± 0.66 nm, polydispersity index of 0.109 ± 0.004, zeta potential of −43.1 ± 1.07 mV, deformability index of 1.254 ± 0.02, and entrapment efficiency of 97.77% ± 0.09%. Transmission electron microscopy revealed that the transfersomes had spherical and unilamellar vesicles. The skin penetration of rhEGF was enhanced by as much as 5.56 fold by transfersomal emulgel compared with that of non-transfersomal emulgel. The stability study illustrated that the rhEGF levels after 3 months were 84.96–105.73 and 54.45%–66.13% at storage conditions of 2°C–8°C and 25°C ± 2°C/RH 60% ± 5%, respectively.

***Conclusion:*** The emulgel preparation containing transfersomes enhanced rhEGF penetration into the skin, and skin penetration was improved by increasing the lipid content.

## Introduction

Epidermal growth factor (EGF) is an endogenous peptide that promotes the processes of cell growth, proliferation, and differentiation.^1^ EGF was first isolated from the submaxillary glands of adult male rats by Cohen et al in 1962.^2,3^ Along with the advancement of biotechnology, recombinant human epidermal growth factor (rhEGF) can be mass-produced from *Escherichia coli* , which has accelerated the development of EGF formulations for healing skin conditions such as chronic wounds, burns, and diabetic ulcers.^1,4,5^

Recently, rhEGF has also been used for cosmetic purposes, such as hiding scars and reducing signs of skin aging.^1^ The method of delivery becomes important if rhEGF is used as a cosmetic because the drug must pass through the stratum corneum, the main skin barrier composed of a layer of keratinocytes.^6,7^

Structurally, rhEGF is a polypeptide chain consisting of 53 amino acid residues with a molecular weight of 6045 Da, and it possesses three disulfide bonds.^8^ Conversely, the ideal characteristics of a drug for skin delivery include a relatively low molecular weight (<500 Da) and melting point (<200°C), moderate lipophilicity (log P 1–3) and aqueous solubility (>1 mg/mL), and high pharmacological potency.^6^ Because of its relatively large molecular weight, it can be difficult for rhEGF to penetrate the skin.

One strategy for increasing the penetration of rhEGF into the skin is encapsulation in vesicles such as liposomes. Jeon et al^9^ successfully encapsulated rhEGF into liposomes for topical delivery. Their results indicated that liposomes can increase the permeation and localization of rhEGF in the skin. However, conventional liposomes generally tend to accumulate in the stratum corneum, the upper skin layer, with minimal penetration into deeper tissues.^10^

In a recent study, lipid-based vesicles known as elastic (flexible) liposomes, also termed ultradeformable vesicles (UDV), were developed in the early 1990s. Transfersomes are UDVs that exhibit great ability to penetrate the skin by passing through pores in the stratum corneum and delivering the drug to the epidermis and dermis.^11^ The flexibility of the transfersome membrane is achieved by mixing phospholipids and an edge activator at proper ratios.^12^ Transfersomes also can encapsulate molecules with various solubility properties and can protect drugs against metabolic degradation.^13^

For the ease of use, transfersomes can be formulated into semisolid dosage formulations such as emulgel. Emulgel is an emulsion, either water-in-oil or oil-in-water, which is incorporated into a gel base. Because of its non-greasy properties, emulgel can be used more comfortably on the skin compared with other topical formulations such as creams and ointments.^14,15^

In this study, rhEGF was encapsulated into transfersomes as a carrier to increase its permeation into the skin. The obtained rhEGF transfersomes were characterized, including analyses of particle size distribution, polydispersity, zeta potential, entrapment efficiency, and deformability. Then, rhEGF transfersomes were formulated in emulgel for topical preparations. Given that rhEGF is a protein peptide that is susceptible to oxidation, deamidation, and aggregation, this polypeptide formulation is a challenge.^16^ The instability of protein peptides in the formulation will damage the appearance of the product and reduce its purity, potential, and pharmacological effects.^17^ Finally, to examine its permeability into skin, the rhEGF transfersomal emulgel was tested for penetration *in vitro* using Franz diffusion cells. This study aimed to prepare and characterize transfersomal emulgel containing rhEGF and the enhancement of skin penetration compared with the findings for non-transfersomal rhEGF emulgel.

## Material and Methods

### Materials

rhEGF used in this study was kindly donated by Daewoong Pharmaceutical Co. Ltd. (Seoul, South Korea). Phospholipon 90G was a gift from Lipoid GmbH (Kӧln, Germany). Sodium deoxycholate and butylated hydroxytoluene were purchased from Sigma-Aldrich (St. Louis, Missouri, USA) and Sterlitamak Petrochemical Plant (Sterlitamak, Russia), respectively. Potassium dihydrogen phosphate and sodium hydroxide were obtained from Merck (Darmstadt, Germany). Sepigel 305 (Seppic, Paris, France), propylene glycol (Qingdao Aspirit Chemical Co. Ltd, Qingdao, China), Na_2_EDTA (Amresco, Ohio, USA), methylparaben (Clariant, Höchst,Germany), and propylparaben (Clariant, Pontypridd, UK) were of pharmaceutical grade. All other solvents and reagents were of analytical grade.

### Methods

#### Preparation of rhEGF -loaded transfersomes

The transfersomes were generated using thin-film hydration followed by extrusion. The formulation is presented in Table 1. First, the phospholipid, edge activator, and antioxidant (butylated hydroxytoluene) were dissolved in ethanol and placed in a round-bottom flask. The solution then was evaporated using a rotary vacuum evaporator (Buchi V-100, Switzerland) at 150 rpm and 40°C under vacuum. After the thin layer was formed, it was streamed with nitrogen gas and stored in the refrigerator overnight to allow complete evaporation of the solvent. rhEGF was incorporated along with 0.2 M phosphate buffer solution (pH 7.2) in the hydration process. Hydration of dry lipid film was performed at 50–250 rpm and 37°C for 45 min. The resulting transfersome suspension was extruded 11 times through polycarbonate membranes (200 nm).^18^

**Table 1:** Formulation of the recombinant human epidermal growth factor (rhEGF) transfersomes

**Material**	**Concentration (% w/v)**		
	**TF-EGF1 (200:1)**	**TF-EGF2 (133:1)**	**TF-EGF3 (100:1)**
rhEGF	0.0250	0.0375	0.0500
Phospholipon 90G	4	4	4
Sodium deoxycholate	1	1	1
Butylated hydroxytoluene	0.5	0.5	0.5
pH 7.2 phosphate buffer	up to 100	up to 100	up to 100

TF-EGF: rhEGF loaded transfersome.

#### Characterization of rhEGF -loaded transfersomes

##### Particle size and zeta potential analysis

The particle size distribution and zeta potential of the formulations were determined using Zetasizer ZS90 (Malvern, UK) in triplicate. The size distribution was expressed as the mean hydrodynamic diameter (Z_average_) and polydispersity index. The transfersome suspension was diluted 40-fold with distilled water. Vesicular size and the polydispersity index were determined using the dynamic light scattering method.^19^

##### Entrapment efficiency

The entrapment efficiency of rhEGF was determined using the centrifugation method. Transfersomes were diluted 7-fold with phosphate buffer solution (pH 7.2) and then centrifuged at 13,000 rpm and 4°C for 3 h. The supernatant, which contained untrapped drug, was carefully collected, diluted with phosphate buffer solution (pH 7.2), and assayed using a human EGF enzyme-linked immunosorbent assay (ELISA) kit. The entrapment efficiency of rhEGF in transfersomes was determined using the following equation:

(1) $$EF(\%)=\frac{Q_{T}-Q_{S}}{Q_{T}}.100\%$$

where EE (%) is the entrapment efficiency, Q_T_ is the total concentration of rhEGF in the initial formulation (μg/mL), and Q_S_ is the concentration of untrapped rhEGF (μg/mL).^9,20^

##### Morphology of vesicles

Vesicle morphology was observed using transmission electron microscopy (TEM, Microscope Tecnai 200 kV D2360 SuperTwin, Thermo Fisher Scientific, USA) with an accelerating voltage of 80 kV. An aliquot (5 μL) of the transfersome suspension was placed on a carbon-coated grid. Excess solution was carefully removed using filter paper. The sample was observed at a magnification of ×29 000–145 000.^9^

##### Deformability index

In total, 1 mL of the transfersome suspension was extruded using a polycarbonate membrane with a pore size of 100 nm in a mini-extruder set (Avanti Polar Lipids Inc., USA). The extruded suspension volume in 5 min was recorded, and the particle size was then determined using the dynamic light scattering method. The deformability index was calculated using the following equation:

(2) $$D=J{\left(\frac{rv}{rp}\right)}^{2},$$

where *D* is the deformability index, *J* is the amount of transfersome suspension that passed through the membrane in 5 min (mL), *rv* is the particle size of the transfersomes that passed through the membrane (nm), and *rp* is the membrane pore size (nm).^21^

#### Emulgel preparation

The composition of gel formulations containing rhEGF is described in Table 2. The emulgel was made by allowing Sepigel 305 to swell in the water containing Na_2_EDTA as a chelating agent. After that, the solution of methylparaben and propylparaben in propylene glycol was added to make the emulgel base. Finally, the rhEGF-loaded transfersomes or rhEGF solution was added to the emulgel base and stirred homogeneously. The formulations were evaluated for organoleptic properties, viscosity, rheology, pH, and rhEGF content.

**Table 2:** Formulation of the recombinant human epidermal growth factor (EGF) emulgel

**Material**	**Concentration (% w/w)**			
	**ETF1**	**ETF2**	**ETF3**	**ENTF**
rhEGF-loaded transfersomes	TF-EGF1 equal to 0.001 rhEGF	TF-EGF2 equal to 0.001 rhEGF	TF-EGF3 equal to 0.001 rhEGF	-
rhEGF solution	-	-	-	Equal to 0.001 rhEGF
Sepigel 305	3	3	3	3
Na_2_EDTA	0.05	0.05	0.05	0.05
Propylene glycol	7	7	7	7
Methylparaben	0.1	0.1	0.1	0.1
Propylparaben	0.05	0.05	0.05	0.05
Distilled water	Up to 100	Up to 100	Up to 100	Up to 100

ETF: rhEGF-loaded transfersomal emulgel; ENTF: rhEGF-loaded nontransfersomal emulgel; TF-EGF: rhEGF-loaded transfersome.

#### In vitro penetration test

The skin penetration properties of emulgel containing rhEGF-loaded transfersomes and rhEGF solution were evaluated *in vitro* using Franz diffusion cells. The membrane used was the abdominal skin of 8–10-week-old female Sprague–Dawley rats weighing approximately 200 g. The skin was mounted onto the diffusion chamber with the stratum corneum and dermal side facing the donor and receptor compartments, respectively. The diffusion area was 1.77 cm^2^. The receptor compartment was filled with 15 mL of phosphate buffer solution (pH 7.4) kept at a temperature of approximately 32°C, and the contents were stirred with a magnetic bar at 300 rpm. A 1-g sample was applied to the donor compartment. Then, 1 mL of sample was withdrawn at predetermined intervals of 1, 1.5, 2, 4, 6, 8, 10, and 12 h, and the same volume of fresh buffer was added to the receptor compartment. The amount of rhEGF penetrated was evaluated using a human EGF ELISA kit. The permeability coefficient (*K*_p_) was calculated using the following equation:

(3) $$K_{p}=\frac{J_{s}}{C_{d}}(cm/h),$$

where *J*_s_ is the flux calculated at steady state (ng/cm^2^·h^1^) and *C*_d_ is the concentration of drug in the donor compartment (ng/cm^3^).

#### Stability of rhEGF -loaded transfersomal emulgel

For products intended for refrigerated storage, the conditions for the stability study were 5°C ± 3°C for the long-term stability, and 25°C ± 2°C with RH 60% ± 5% for the accelerated stability study.^22^ In this study, the samples were stored for 3 months and evaluated monthly for appearance, viscosity, pH, and rhEGF content.

#### Quantification of rhEGF

rhEGF levels were quantified using an ELISA kit for rhEGF (Human EGF ELISA kit, Pink-ONE, Komabiotech, Seoul, South Korea) according to the manufacturer’s procedures. Aliquots of samples were diluted with phosphate buffer solution (pH 7.2), added to an antibody-coated ELISA plate together with standards, and incubated at room temperature for 2 h. Diluted detection antibody solution, diluted streptavidin-horseradish peroxidase solution, and tetramethylbenzidine reagent were stepwise added and incubated according to the kit procedures. The reaction was stopped by the addition of H_2_SO_4_ stop solution, and the absorbance was read immediately at 450 nm using a VersaMax microplate reader (Molecular Devices LLC, California, USA). A standard curve was obtained using standard rhEGF provided in the ELISA kit within the concentration range of 4–250 pg/mL.

#### Statistical analysis

rhEGF immunoassay was calculated using SoftMax Pro version 7.0.3 (Molecular Devices LLC, California, USA). Data were expressed as a mean value ± standard deviation (SD). Statistical analysis was performed using *t* test by Microsoft Office Excel version 16.30 to analyze the results of *in vitro* penetration study of rhEGF (n = 2). A *P* value of less than 0.05 was considered to be significant.

## Results and Discussion

### Preparation of rhEGF-loaded transfersomes

Excipients and the manufacturing condition were carefully chosen to maintain the stability of the active substance rhEGF because of its susceptibility to oxidation, deamidation, aggregation, heat, and acid/base reactions.^16,23,24^ The selected buffering agent was 0.2 M phosphate buffer solution (pH 7.2) because it is known to minimize the aggregation of rhEGF over the pH range of 6.0–8.0, especially at pH near 7.2.^16,17,23,25^ Furthermore, phosphate buffer 0.2 M has a higher buffering capacity than other types of buffering agents, in addition to being more cost-effective and more widely used in protein formulation.^16^ Sodium deoxycholate was selected because its high hydrophilic-lipophilic balance (HLB = 16); therefore, it can encapsulate hydrophilic drugs, such as rhEGF, more efficiently than low-HLB surfactants.^26^ Other surfactants with high HLB values, such as polysorbate 80 (HLB = 15), were avoided because they can auto-oxidize to protein-damaging peroxides and reactive aldehydes in aqueous solution.^27^ Santana et al^16^ also reported that polysorbate 80 increased the Met-21 oxidation of rhEGF. During the manufacturing process, the temperature was maintained below the unfolding point of 40°C because of the poor thermal stability of rhEGF.^25^

The selected concentrations of phospholipid and edge activator in the transfersome vesicle preparation were based on our earlier findings.^18^ We determined that the formulation with a phosphatidylcholine:sodium deoxycholate ratio of 80:20 was superior given its characteristics such as spherical and unilamellar vesicles, a particle size of less than 200 nm, a polydispersity index close to 0, a zeta potential smaller than −30 mV, and good elasticity and deformability. In this present study, we encapsulated rhEGF at several concentrations into the selected optimized formulation. The concentration of 0.025% rhEGF in formula TF-EGF1 was based on a study by Jeon et al,^9^ who encapsulated rhEGF into a liposomal formulation at that concentration. Furthermore, the rhEGF concentration was increased in formulas TF-EGF2 and TF-EGF3 to study the effect on the entrapment efficiency.

### Characterization of rhEGF-loaded transfersomes

The characteristics of the three formulations of rhEGF-loaded transfersomes are summarized in Table 3.

**Table 3:** Characteristics of recombinant human epidermal growth factor-loaded transfersomes

**Formulation**	**Particle size (nm)**	**PDI**	**Zeta Potential (mV)**	**Entrapment Efficiency (%)**
TF-EGF1	128.1 ± 0.66	0.109 ± 0.004	-43.1 ± 1.07	97.77 ± 0.09
TF-EGF2	125.4 ± 0.61	0.110 ± 0.008	-36.8 ± 2.08	92.78 ± 2.11
TF-EGF3	118.7 ± 1.11	0.116 ± 0.007	-40.5 ± 0.90	92.15 ± 0.38

PDI: polydispersity index; TF-EGF: rhEGF-loaded transfersome. Data are presented as the mean ± standard deviation (n = 3).

#### Particle size and zeta potential

The dynamic light scattering method measures the Brownian motion of particles, which is related to the particle size.^19^ In dynamic light scattering, Z_average_ is the most important and stable variable associated with the technique.^28^ Z_average_ is an intensity-weighted distribution in which the contribution of each particle to the distribution is related to the light scattered by the particle.^29^ In this measurement, the polydispersity index is also an important parameter that reflects the particle width. This value ranges from 0 to 1, with a smaller value indicating a more homogeneous particle size distribution.^19^

**Figure 1:**
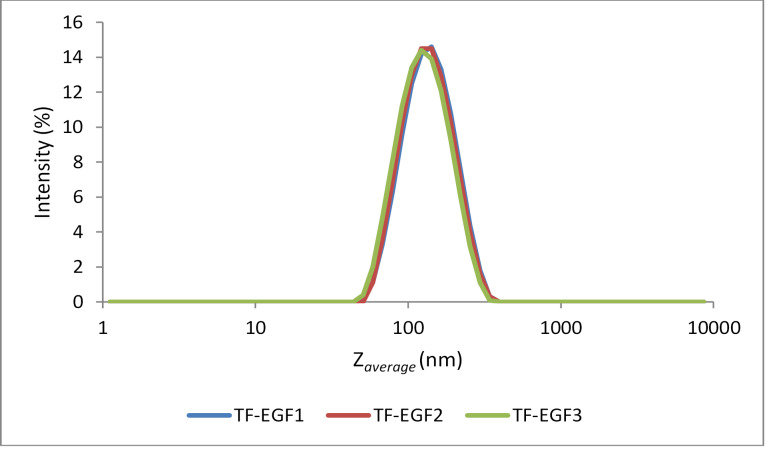
The particle size distribution of the recombinant human epidermal growth factor-loaded transfersomes.

All formulations had a particle size of less than 200 nm according to the pore size of the polycarbonate membrane used in the extrusion step. As shown in Figure 1, the particle size distributions of all rhEGF-loaded transfersomes formulations were similar. Furthermore, the addition of rhEGF to the transfersomes did not alter the particle size distribution, as the particle size distributions of TF-EGF1–TF-EGF3 were similar to that of the transfersome formulation without rhEGF (data not shown). It appears that the particle size distribution remained the same because the vesicle concentration greatly exceeded the added the rhEGF concentration. The polydispersity indices of the three formulations were satisfactorily less than 0.2. PDI is dimensionless with the numerical value of PDI ranges from 0.0 (for a perfectly uniform sample with respect to the particle size) to 1.0 (for a highly polydisperse sample with multiple particle size populations). In drug delivery applications using lipid-based carriers, a PDI of 0.3 and below is considered to be acceptable and indicates a homogenous population of phospholipid vesicles.^30^

In addition to the particle size distribution and polydispersity index, zeta potential was also measured using the same tools. Zeta potential is a measure of the magnitude of the electrostatic or charges repulsion or attraction between particles in a liquid suspension. Zeta potential is a fundamental parameter for describing the stability of a dispersion system, as it provides detailed insight into the causes of dispersion, aggregation, or flocculation.^29^

All formulations of rhEGF-loaded transfersomes had zeta potential values of less than −30 mV, indicating good stability.^28^ The causes of the differences in zeta potential among the formulations are unclear because there was no linear correlation between the rhEGF concentration and the zeta potential. The net negative charge resulted from the lipid and edge activator in the formulations. Phosphatidylcholine has an isoelectric point between 6 and 7. During the manufacturing process, phosphate buffer solution (pH 7.2) was added as the hydrating medium. Because the pH was slightly higher than the isoelectric point of phosphatidylcholine, phosphatidylcholine carried a negative charge.^21,31^ Moreover, the edge activator (sodium deoxycholate) is an anionic surfactant that also contributed the net negative charge of the formulations.^26^

#### Entrapment efficiency

In this study, we achieved entrapment efficiencies exceeding 90% for all formulations. This might be because of the small concentration of rhEGF used (0.025%–0.050%) in comparison with that of the vesicle in the formulation. Furthermore, the entrapment efficiency usually depends on the concentration of the lipid used. In a prior study, the entrapment efficiency increased with higher concentrations of phosphatidylcholine.^20^ As evidenced in Table 3, TF-EGF1, with the highest ratio of lipid to the active substance (200:1), displayed the highest entrapment efficiency. A similar result also obtained in a study conducted by Yang et al,^32^ who found that a smaller dosage of terbinafine HCl (higher ratio of lipid to the active substance) resulted in the highest entrapment efficiency among all transfersome formulations. In addition, the HLB of the edge activator used appositively contributed to the entrapment efficiency. Bnyan et al^26^ mentioned that surfactants with high HLB values, such as sodium deoxycholate, tend to increase the entrapment efficiency of hydrophilic drugs. This was proved by Shaji et al,^33^ who obtained higher entrapment efficiency for piroxicam-loaded transfersomes with sodium deoxycholate than with Span 65, Span 80, and Tween 80. Ternullo et al,^34^ who prepared anionic deformable liposomes containing human EGF with sodium deoxycholate, obtained an entrapment efficiency of 84.1% ± 6.1%.

#### Morphology of vesicles

**Figure 2:**
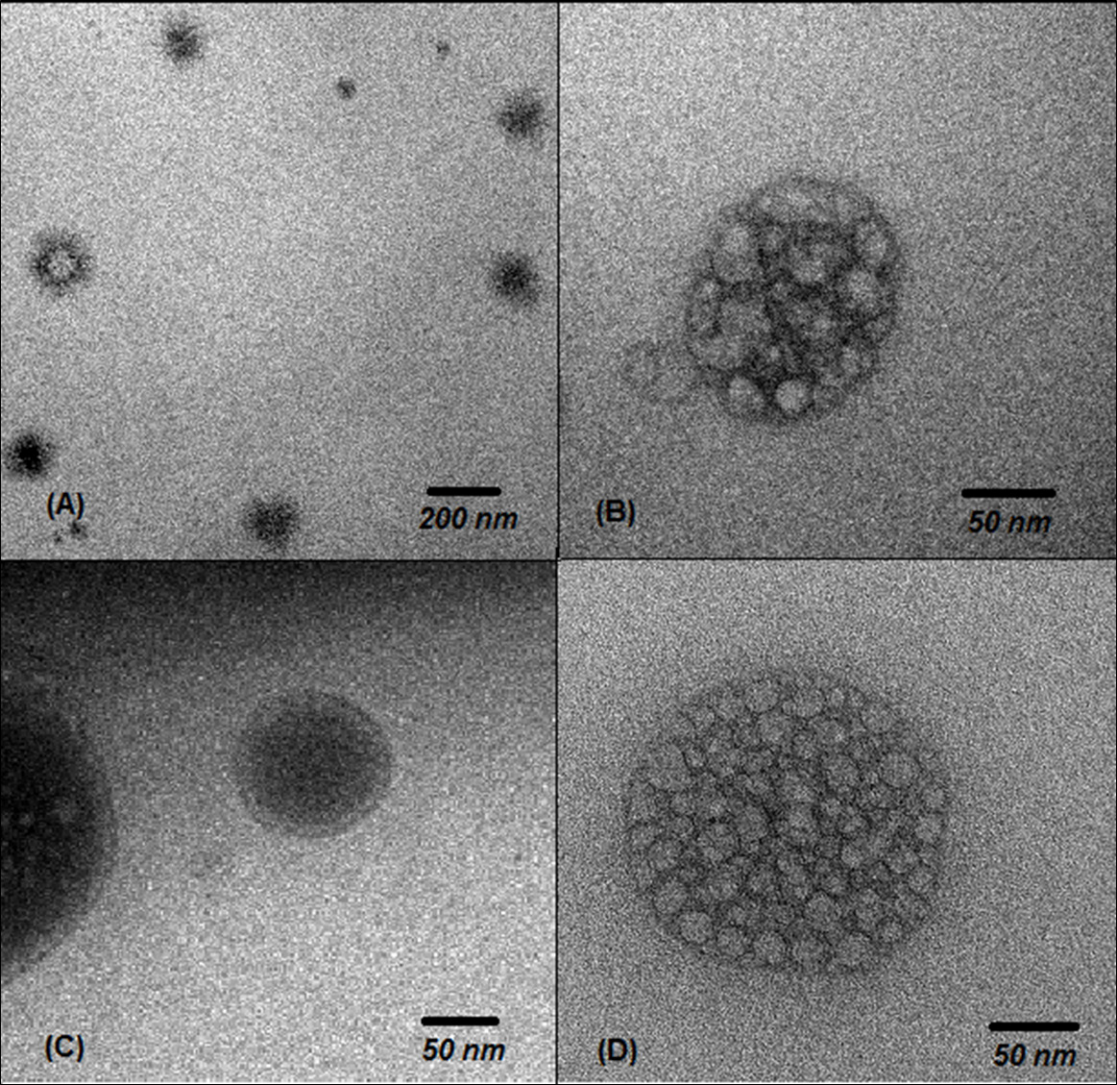
Transmission electron micrographs of recombinant human epidermal growth factor-loaded transfersomes. (a) TF-EGF1, ×29 000 magnification; (b) TF-EGF1, ×145 000 magnification; (c) TF-EGF2, ×71 000 magnification; (d) TF-EGF3, ×145 000 magnification.

TEM of the rhEGF-loaded transfersomes, as shown in Figure 2, confirmed that the obtained vesicles were spherical and unilamellar. There was no disruption of vesicular structure even after the application of various mechanical stresses such as extrusion, and good vesicle integrity ensures that no leakage of active substances from the vesicles occurs.^35^ As presented in Figure 2, the vesicle sizes were approximately 120, 160, and 190 nm for TF-EGF1, TF-EGF2, and TF-EGF3, respectively. The size of TF-EGF1 vesicles was similar to Z_average_. Conversely, the vesicle sizes of TF-EGF2 and TF-EGF3 exceeded Z_average_, indicating that the transfersome images captured particles in the range of d_v_90%.

#### Deformability index

The deformability index was used to examine the flexibility of transfersomes. Vesicles with better membrane deformability could penetrate the lipid membrane through hydrophilic pathways or pores between the cells that are smaller than their diameter without losing their vesicle integrity. This becomes possible by incorporating an edge activator that destabilizes the lipid bilayer and increases the fluidity and elasticity of the vesicles.^36^

The deformability index data are presented in Table 4. All rhEGF-loaded transfersome formulations were deformable because they could pass through the membrane entirely. The data illustrated that the deformability indices of the three rhEGF transfersome formulations were similar to that of the transfersomes formula lacking rhEGF (data not shown).^18^ Changes in particle size after extrusion did not occur in this test, proving that the vesicles maintained their integrity even after passing through smaller pores.

**Table 4:** Deformability indices of recombinant human epidermal growth factor-loaded transfersome formulations

**Formulation**	**Particle size after extrusion (nm)**	**Deformability index**
TF-EGF1	112.0±1.00	1.254±0.02
TF-EGF2	107.9±2.47	1.165±0.05
TF-EGF3	110.7±0.98	1.226±0.02

#### In vitro penetration test

**Figure 3:**
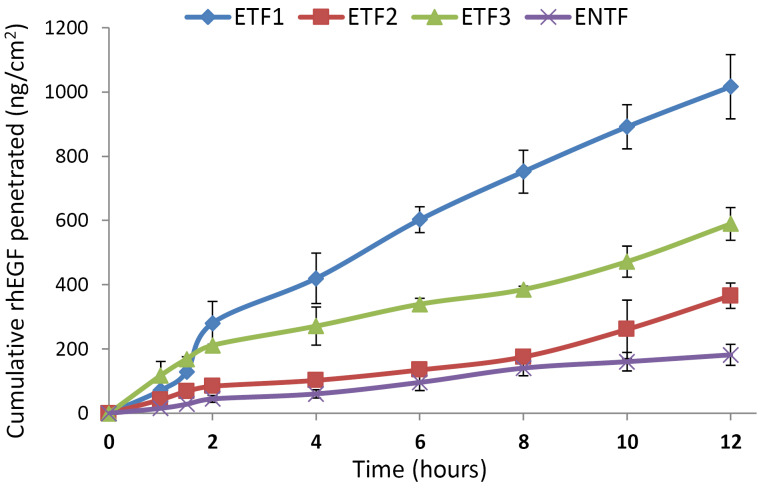
The cumulative amounts of recombinant human epidermal growth factor (rhEGF) penetrated from emulgel preparations. Note: *Statistically significant (*P* < 0.05), compared with rhEGF-loaded transfersomal emulgel (ETF1–3).

The penetration study was conducted for 12 h with eight sampling intervals. During the test, the obtained samples were immediately replaced with the same volume of medium to maintain a constant drug concentration in the receptor compartment and form a sink condition.^37^ Figure 3 shows the cumulative amounts of rhEGF that penetrated from the transfersomal emulgel and non-transfersomal emulgel formulations through rat skin over 12 h. From this test, the highest cumulative amount of penetrated rhEGF was obtained for the ETF1 formula, followed by ETF3 and ETF2. The amounts of penetrated rhEGF from the three transfersomal emulgel preparations were significantly (*P* < 0.05) higher than that from the non-transfersomal emulgel formula (ENTF). The ETF1 formula may have had the highest cumulative amount of penetrated rhEGF because the entrapment efficiency of the TF-EGF1 formula also exceeded those of the other two transfersomes formulas. This suggested that the entrapment efficiency of the transfersomes affected the cumulative amount of active substance penetrated. However, the entrapment efficiency was not correlated with the ETF3 penetration result in that this formulation had a higher level of rhEGF penetration than ETF2 despite the similar entrapment efficiencies of TF-EGF2 and TF-EGF3.

Based on the graph of the cumulative amount of penetrated rhEGF (ng) per unit area of diffusion (cm^2^), the flux or rate of drug release (ng/cm^2^·h^1^), permeability coefficient, and enhancement ratio were calculated, as shown in Table 5. Flux was obtained from the slope and taken at steady state according to Fick’s law, whereas the permeability coefficient was calculated using equation 3. From these data, it appeared that the flux and permeability coefficient were highest for the ETF1 formula and lowest for the ENTF formula.

**Table 5:** *In vitro* flux, coefficient permeability, and enhancement ratios of the recombinant human epidermal growth factor emulgel

**Formulation**	**Flux** **(ng/cm** ^ 2 ^ **.h)**	**Kp × 10**^-3^ **(cm/h)**	**Enhancement Ratio**
ETF1	86.003 ± 6.108	8.600 ± 0.611	5.56
ETF2	26.026 ± 4.587	2.603 ± 0.459	1.68
ETF3	41.796 ± 2.660	4.180 ± 0.266	2.70
ENTF	15.472 ± 2.831	1.547 ± 0.283	1

Kp, permeability coefficient; ETF, rhEGF-loaded transfersomal emulgel; ENTF, rhEGF-loaded nontransfersomal emulgel. Data are presented as the mean ± standard deviation (n = 2).

The calculated percentages of total rhEGF penetrated from each preparation over 12 h for ETF1, ETF2, ETF3, and ENTF emulgel preparations were 17.99 ± 0.47, 6.48 ± 0.70, 10.43 ± 0.90, and 3.22% ± 0.58%, respectively. These values far exceeded the results reported by Jeon et al.^9^ Their research examined the *in vitro* penetration of liposomes containing 250 μg of rhEGF into rat skin over 12 h. The results indicated that the percentage of total rhEGF penetrated was approximately 1.36% with a flux of 37.75 ± 21.80 ng/cm^2^·h^1^. In that study, rhEGF was mostly deposited in rat skin.^9^ A smaller flux value was also obtained by Lu et al,^38^ who performed *in vitro* penetration tests of EGF cationic deformable liposomes (CDLs) and EGF CDL ointments using rat skin. The obtained flux values obtained in these studies were 0.45 and 0.18 ng/cm^2^·h^1^ for EGF CDLs and EGF CDL ointments, respectively.^38^ Meanwhile, in the *ex vivo* penetration test conducted by Ternullo et al^34^ using full-thickness human skin and Franz diffusion cell systems, no EGF penetrated through the skin over 6 h after the delivery of deformable liposomes. From these data, the rhEGF-loaded transfersomes developed in this present study could significantly increase the delivery of rhEGF through the skin compared with the findings for non-transfersome formulations and the results of other similar studies. This proved that formulation into transfersomes increases the penetration of active substances into the skin.

Some studies of the transferosomal emulgel ability for skin permeation enhancement have been reported using similar molecular weight molecules with rhEGF, such as insulin.^39,40^ Malakar et al have conducted the enhancement of insulin (6 kDa) delivery into the skin using cholate-based transfersomal gel by iontophoretic influence (with 0.5 mA/cm^2^ current supply) provided further enhancement of permeation flux.^39^ Another study reported that the insulin transferosomal gel with chemical penetration enhancer showed better glucose lowering effect as compared to the control gel.^40^

There are two possible mechanisms of action by which transfersomes can increase the delivery of drugs through the skin. First, vesicles can act as drug-carrying systems that completely pass through the stratum corneum while carrying drug molecules into the skin.^7,41^ Transfersomes are UDVs with more elastic properties than conventional liposomes because they can change their shape when delivering drugs through the membrane. The ability of transfersomes to deform is attributable to the presence of a surfactant component that acts as an edge activator that destabilizes the lipid bilayer and increases the deformability of vesicles.^10,11^ Transfersomes have sufficient flexibility to pass through pores with much smaller diameters (up to one-tenth of the diameter of the transfersomes).^6^ The driving force for penetration into the skin is an osmotic gradient caused by differences in water content between the surface of the skin, which is relatively dehydrated (approximately 20% water), and the more watery epidermis.^42^ Deformable transfersomes can enter through a narrow gap in the stratum corneum lipids and then penetrate deeper following the osmotic gradient.^10,43^ In the second mechanism, vesicles can act as penetration enhancers primarily through the modification of intercellular lipid lamellae caused by the entry of bilayer vesicles into the stratum corneum. This will facilitate the penetration of free drug molecules into the stratum corneum.^41^

Moreover, emulgel preparation was more beneficial, as emulgel helps in the incorporation of hydrophobic moiety, such as lipid nanovesicle, into the oily globules dispersed in aqueous phase resulting in oil/water emulsion. The emulsion can be mixed into gel base. This may prove better stability and release of drug than simply incorporating drugs into gel base.^44^

#### Stability study

**Figure 4:**
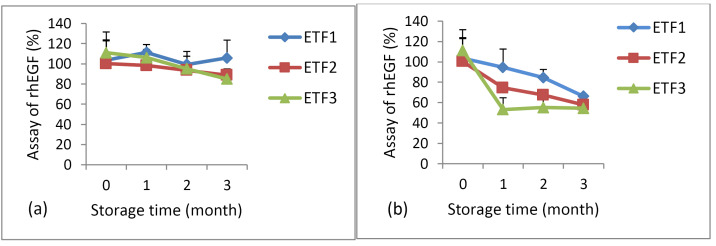
Stability data of recombinant human epidermal growth factor-loaded transfersomal emulgel under storage at (a) 5°C ± 3°C and (b) 25°C ± 2°C/RH 60% ± 5% for 3 months.

The results of the stability study during storage at 5°C ± 3°C and 25°C ± 2°C/RH 60% ± 5% for 3 months revealed no significant changes in the appearance, viscosity, and pH of the rhEGF-loaded transfersomal emulgel preparations (data not shown). The stability of rhEGF content also met the criterion (80%–120%) for ETF1, ETF2, and ETF3 during storage at 5°C ± 3°C (Figure 4a). Meanwhile, during storage at 25°C ± 2°C/RH 60% ± 5% for 3 months, the ETF1 formulation had the best stability, with an rhEGF content of 66.13% ± 0.82%. Meanwhile, the rhEGF content decreased to 57.79% ± 1.44% for ETF2 and 54.45% ± 3.84% for ETF3 in the third month (Figure 4b). This occurred because of the rhEGF protein is unstable at temperatures exceeding 4°C, indicating that rhEGF is a thermolabile protein.

## Conclusion

In this study, rhEGF-loaded transfersomes were successfully produced using the thin-film hydration-extrusion method. The best formulation of transfersomes was obtained with a vesicle:active ingredient ratio of 200:1, which produced spherical and unilamellar vesicles of less than 200 nm in diameter with high homogeneity, good zeta potential, deformability, and high entrapment efficiency. The skin penetration of rhEGF was enhanced by transfersomal emulgel compared with the findings for non-transfersomal emulgel. Stability testing also indicated that the rhEGF-loaded transfersomal emulgel was stable during storage at 2°C–8°C for at least 3 months. These results also indicate that the rhEGF-loaded transfersomal emulgel has potential applicability as an anti-aging cosmetic.

## Ethical Issues

All animal treatment was conducted in accordance with the National Institutes of Health (NIH) Guide for the Care and Use of Laboratory Animals. It was also approved by the Ethics Committee of Faculty of Medicine, Universitas Indonesia (No. KET-III/UN2.F1/ETIK/PPM.00.02/2019).

## Conflict of Interest

The authors declare that there is no conflict of interest regarding the publication of this article.

## Acknowledgments

The authors gratefully acknowledge Universitas Indonesia for the 2019 Q1Q2 research grant NKB-0202/UN2.R3.1/HKP.05.00/2019. In addition, we thank PT. Daewoong Pharmaceutical Company Indonesia for providing rhEGF material and rhEGF immunoassay.
